# Liver chemistry in new-onset Henoch-Schönlein syndrome

**DOI:** 10.1186/s13052-017-0405-5

**Published:** 2017-09-21

**Authors:** Giulia Rosti, Gregorio P. Milani, Emanuela A. Laicini, Emilio F. Fossali, Mario G. Bianchetti

**Affiliations:** 1 Pediatric Emergency Unit, Fondazione IRCCS Ca’ Granda Ospedale Maggiore Policlinico, Milan, Italy; 2 Pediatric unit, Department of Clinical Sciences and Community Health, Università degli Studi di Milano, Fondazione IRCCS Ca’ Granda Ospedale Maggiore Policlinico, Via della Commenda 9, 20121 Milan, Italy; 3 Pediatric Department of Southern Switzerland, Bellinzona, Switzerland

**Keywords:** Vasculitis, Allergic purpura, Hepatitis, Elevated liver enzymes, Diagnostic test

## Abstract

**Background:**

Henoch-Schönlein syndrome is a systemic small-vessel leukocytoclastic vasculitis that usually present with cutaneous, gastrointestinal, articular and renal manifestations. Little is known on liver involvement in this syndrome. This study investigated liver chemistry and creatine kinase in Henoch-Schönlein children.

**Case presentation:**

Alanine aminotransferase, aspartate aminotransferase, γ-glutamyltransferase, lactate dehydrogenase, total bilirubin, prothrombin time and creatine kinase were assessed in 75 consecutive pediatric patients (41 boys and 34 girls aged from 2.9 to 17 years) with new-onset Henoch-Schönlein syndrome. Mildly altered values were found in 7 (9%) patients (5 boys and 2 girls aged from 3.3 to 17 years). In the mentioned cases, all tests returned to normal at a 2–4-week follow-up.

**Conclusions:**

This preliminary study points out that altered and self-remitting liver parameters occur in approximately 10% of children with Henoch-Schönlein syndrome.

## Background

Henoch-Schönlein syndrome, also called immunoglobulin A vasculitis, is the most common systemic vasculitis in childhood [1]. It is a small-vessel leukocytoclastic vasculitis that typically presents with cutaneous, gastrointestinal, articular and renal manifestations [1]. Little is known on liver involvement in this syndrome. Limited data suggest that aminotransferases are elevated in approximately 3% of children with new-onset Henoch-Schönlein syndrome and in cases complicated by acalculous cholecystitis or pancreatitis [2, 3]. Exercise, infectious prodromi or drugs with hepatotoxic potential or a pre-existing liver disease, such as non-alcoholic fatty liver disease, might also alter aminotransferases in Henoch-Schönlein syndrome [4, 5]. Herein, we report our prospective single-center experience with liver chemistry and creatine kinase in 75 consecutive pediatric patients affected by this vasculitis syndrome.

## Study population and methods

Starting in 2009, at the Pediatric Emergency Unit, Fondazione IRCCS Ca′ Granda Ospedale Maggiore Policlinico, Milan, Italy, the initial laboratory approach to new-onset Henoch-Schönlein cases included among others the determination of liver chemistry (alanine aminotransferase, aspartate aminotransferase, γ-glutamyltransferase, lactate dehydrogenase, total bilirubin and prothrombin time) and creatine kinase in plasma. Urinalysis for hematuria and proteinuria was also assessed.

In subjects with abnormal enzyme levels, plasma amylase was determined and a hepatobiliary ultrasound performed. Finally, the blood tests were repeated approximately 2–4 weeks later.

The diagnosis of Henoch-Schönlein syndrome was made using the Ankara 2008 - EULAR/PRINTO/PRES criteria [6]. The **CAAR** grading system for **c**utaneous, **a**bdominal, **a**rticular, and **r**enal involvement was applied to assess the systemic disease activity [7].

From each case, we collected data on ethnicity, pre-existing chronic conditions, possible triggers (defined as acute illness or vaccination [1, 8] preceding the skin lesions by ≤14 days) and repetitive exercise ≤3 days before admission to the emergency department. A careful drug history, including both prescribed and over-the-counter medications, was also taken.

The liver and spleen borders were estimated clinically at the end of inspiration: the liver border was determined by percussion and palpation in the right mid-clavicular line and the diagnosis of hepatomegaly was made when the liver edge projected >2 cm below the margin [9]; the spleen border was determined by palpation in the left anterior axillary line and the diagnosis of splenomegaly was made when the spleen was palpable [10]. Finally, the Murphy sign was considered positive in cases with pain on inspiration while palpating the right upper abdominal quadrant.

A kinetic assay was used for alanine aminotransferase (reference: <50 U/L), aspartate amintransferase (reference: <50 U/L) and amylase (reference: <120 U/L), an ultraviolet spectrophotometry for lactate dehydrogenase (reference: <500 U/L) and creatine kinase (reference: <150 U/L), a kinetic colorimetry for γ-glutamyltransferase (reference: <50 U/L), a diazo colorimetry for total bilirubin (reference: <20 μmol/L) and an automated clotting assay for prothrombin time (reference: 0.90–1.30 INR). An automated dipstick system was used for hematuria and proteinuria.

Enzyme values were graded as normal or mildly (<5 times the upper reference limit), moderately (5–10 times) and markedly (>10 times) altered. Data analysis was approved of by the Ethical Committee of our hospital**.** Anonymized patient information was used for data analysis. Data are given either as median and interquartile range or as relative frequency. The one-sample Mann–Whitney–Wilcoxon test was used to compare the results with those of the general population. Significance was assumed when *P* < 0.05.

## Case presentation

Between January 2009 and June 2016, we made the diagnosis of new-onset Henoch-Schönlein syndrome in 72 Caucasian and 3 Hispanic patients up to 17 years of age presented to the Pediatric Emergency Unit. History, clinical findings and renal involvement are given in Table 1. A possible trigger, either an infection or a vaccination, was reported by 49 (65%) patients. Forty-one (55%) cases had been managed with paracetamol, a nonsteroidal anti-inflammatory drug, a penicillin or a macrolide.

**Table 1:** History, clinical findings and renal involvement in 75 pediatric patients aged from 2.9 to 17 years with new-onset Henoch-Schönlein syndrome. Data are given either as relative frequency or as median and interquartile range

Age, years	6.4 [4.8–7.7]
Gender, ♂: ♀	41: 34
Pre-existing chronic condition, N	0
Recent repetitive exercise	5
Triggers	
Acute lower respiratory infection, N	15
Acute upper respiratory infection, N	14
Nonspecific febrile illness, N	10
Urinary tract infection, N	6
Acute diarrheal disease, N	3
Vaccination, N	1
Drug management	
Paracetamol, N	31
Nonsteroidal anti-inflammatories, N	12
Penicillins, N	15
Macrolides, N	4
Clinical findings	
Hepatomegaly, N	2
Splenomegaly, N	1
Hepatosplenomegaly, N	1
Murphy sign, N	0
Cutaneous involvement	
Mild, N	47
Moderate, N	25
Severe, N	3
Abdominal involvement	
None, N	28
Mild, N	28
Moderate, N	17
Severe, N	2
Articular involvement	
None, N	14
Mild, N	46
Moderate, N	12
Severe, N	3
Renal involvement	
None, N	45
Mild, N	22
Moderate, N	7
Severe, N	1

**Fig. 1:**
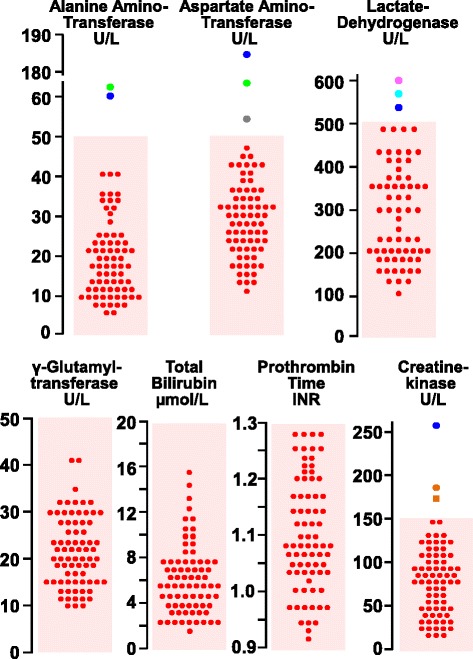
Liver parameters in 75 consecutive children with new-onset Henoch-Schönlein syndrome. An isolated increase in creatine kinase was observed in two boys 7.5 and 9.3 years of age, respectively [orange circle; orange square]. Altered liver parameters were also noted in five subjects: a 5.4-year-old Caucasian male [blue circle], a 6.7-year-old Caucasian male [green circle], a 17-year-old Hispanic female [gray circle], a 6.2-year-old Caucasian male [lavender circle], and a 3.3-year-old Caucasian girl [aqua circle]

The results of liver chemistry and creatine kinase appear in Fig. 1. As compared with the general population, blood values were not abnormally elevated in Henoch-Schönlein patients.

Enzyme levels were normal in 68 and mildly elevated in the remaining 7 patients. None of them presented with hepatomegaly, splenomegaly or Murphy sign. Circulating amylase and hepatobiliary ultrasound were found to be normal in these cases.

An isolated increase in creatine kinase (175 and 189 U/L, respectively) was observed in two boys (7.5 and 9.3 years of age, respectively) with recent history of repetitive exercise. An increase in plasma creatine kinase (265 U/L), associated with elevated alanine aminotransferase (60 U/L), aspartate aminotransferase (186 U/L) and lactate dehydrogenase (523 U/L) was also observed in a 5.4-year-old boy with recent extended physical activity.

An altered liver chemistry was noted in further four patients: a 6.7-year-old Caucasian male (alanine aminotransferase 64 U/L, aspartate aminotransferase 65 U/L); a 17-year-old Hispanic female (aspartate aminotransferase 53 U/L); a 6.2-year-old Caucasian male (lactate dehydrogenase 603 U/L); and a 3.3-year-old Caucasian girl (lactate dehydrogenase level 540 U/L). Henoch-Schönlein syndrome had been preceded by an upper (*N* = 2) or a lower (*N* = 2) respiratory tract infection in these four patients and treatment with paracetamol 40–60 mg/kg body weight daily had been recommended for fever, abdominal pain or arthralgia.

At the 4-week follow-up, the parameters were normal in the 7 patients with altered tests.

## Discussion

This is the first prospective study investigating liver chemistry and creatine kinase in unselected Henoch-Schönlein children. These tests are normal at presentation in about 90% and mildly elevated in 10% of these patients. In the latter cases, liver parameters are transiently altered and self-remit within 4 weeks. Similar observations were recently made in patients affected with acute hemorrhagic edema of young children, the infantile variant of Henoch-Schönlein syndrome [11].

Exercise likely accounts for the altered tests observed in 3 patients with recent history of extended physical activity [4, 5]. On the contrary, we do not have a clear-cut explanation for the transiently altered liver chemistry noted in the remaining 4 cases. We speculate that altered liver tests resulted either from the infectious prodrome or from unreported self-administration of a drug with hepatotoxic potential. Finally, the strategy used to establish cut off values implies that a result out of range is not necessarily pathologic and is found in 2–3% of healthy subjects.

The findings of this study confirm those of a retrospective analysis including apparently unselected Henoch-Schönlein children from the Republic of China [12]. In that report, mildly elevated alanine aminotransferase levels were observed in 9% of the cases. However, creatine kinase levels were not assessed in those children.

Some months after starting the investigation presented in this report, the use of lower laboratory thresholds for alanine aminotranferase (i.e. 25 U/L instead of 50 U/L) was proposed for children affected by a chronic liver disease [13]. This recommendation deserves in our opinion further support.

The main limitations of this preliminary study were its single center design, the rather small sample size and the ethnic homogeneity of the study population.

## Conclusions

This preliminary study points out that altered and self-remitting liver chemistry or creatine kinase are found in approximately 10% of children with Henoch-Schönlein syndrome.
